# Integrated clinico-radio-molecular profiling of diffuse gliomas across age groups: a Romanian single-center cohort study

**DOI:** 10.25122/jml-2026-0050

**Published:** 2026-05

**Authors:** Teodor Cristian Blidaru, Dan Mitrea, Manuela Ghica, Marius Cristian Zaharia, Natalia Blidaru, Maria Sînziana Matei, Raluca Maria Marin, Ioana Raluca Papacocea

**Affiliations:** 1 Carol Davila University of Medicine and Pharmacy, Bucharest, Romania; 2 Neuroaxis Neurology Clinic, Bucharest, Romania

**Keywords:** diffuse gliomas, age-related differences, neuro-oncology, tumor phenotype, survival outcomes

## Abstract

Diffuse gliomas are biologically heterogeneous primary brain tumors with variable clinical behavior, and age is a well-recognized prognostic factor; however, integrated real-world data from Eastern European populations remain limited. The aim of this study was to describe how clinical, imaging, molecular, treatment, and survival data co-vary across age groups in a Romanian tertiary neuro-oncology cohort, rather than to identify new biological associations. We conducted a single-center retrospective cohort study of 283 consecutive adult patients with histologically confirmed diffuse gliomas diagnosed between 2021 and 2024, classified according to the WHO 2021 framework. Patients were stratified into three predefined age groups (<40, 40-60, and >60 years), and clinical, histopathological, molecular, preoperative imaging, and treatment variables were compared using Pearson chi-square or Fisher exact tests; recurrence-free survival (RFS) was estimated using the Kaplan-Meier method with log-rank tests. Molecular testing availability varied across the cohort, reflecting progressive adoption of integrated molecular diagnostics. Older patients more frequently harbored IDH-wildtype glioblastoma, more aggressive imaging features, lower Karnofsky Performance Status, and received less intensive multimodal therapy. Unadjusted RFS decreased with advancing age (log-rank *P* < 0.001); without multivariable adjustment, this reflects the combined effects of age-correlated covariates rather than an independent age effect. This study contributes region-specific real-world evidence from an underrepresented Eastern European setting, where integrated molecular diagnostics were being progressively implemented during the study period.

## INTRODUCTION

Diffuse gliomas represent the most common primary intra-axial brain tumors in adults and remain among the most biologically heterogeneous and clinically challenging entities in neuro-oncology. The 2021 World Health Organization (WHO) Classification of Tumors of the Central Nervous System has redefined these tumors through an integrated histo-molecular framework, in which molecular alterations such as isocitrate dehydrogenase (IDH) mutation status, chromosome 1p/19q codeletion, and cyclin-dependent kinase inhibitor 2A/2B (CDKN2A/B) deletions are now central to diagnostic classification, while O6-methylguanine-DNA methyltransferase (MGMT) promoter methylation is used as a predictive biomarker for therapeutic decision-making rather than as a diagnostic criterion [1,2]. Contemporary guidelines from the European Association of Neuro-Oncology (EANO) further highlight that optimal patient management requires the convergence of clinical, imaging, histopathological, and molecular data within a multidisciplinary setting [1]. Despite these advances, age remains one of the most powerful and consistently reported prognostic factors in diffuse gliomas, influencing tumor biology, therapeutic strategy, and survival outcomes [3,4]. However, real-world data from Eastern European populations remain limited in the literature, particularly large, population-based cohorts.

Under the 2021 WHO classification, adult-type diffuse gliomas are grouped into IDH-mutant astrocytoma, IDH-mutant 1p/19q-codeleted oligodendroglioma, and IDH-wildtype glioblastoma [2]. This integrated histo-molecular framework defines biologically and prognostically distinct entities, each with characteristic clinical trajectories.

Substantial biological heterogeneity persists within diffuse gliomas, and age appears to modulate both tumor genotype and phenotype. Younger patients are more frequently diagnosed with IDH-mutant astrocytomas or oligodendrogliomas, whereas older individuals predominantly present with IDH-wildtype glioblastoma, a subtype associated with more aggressive behavior and poorer prognosis [2,4]. In addition, prior studies have suggested that age influences treatment decisions and tolerance, with elderly patients being less likely to receive aggressive multimodal therapy and more prone to treatment-related toxicity [5].

Imaging features such as contrast enhancement, necrosis, and elevated relative cerebral blood volume (rCBV) are commonly associated with higher-grade IDH-wildtype tumors, which are more prevalent in older populations [6,7]. Large-scale genomic analyses indicate that IDH-mutant and IDH-wildtype gliomas follow divergent molecular trajectories [8,9], and age-related host factors may further modulate tumor–host interactions [10].

Randomized clinical trials in diffuse glioma frequently underrepresent elderly patients and those with reduced performance status [11]. Real-world cohorts are therefore essential to understanding how age shapes diagnostic workup, treatment allocation, and survival outside trial settings [11,12]. Similarly, real-world cohort studies have increasingly explored how key demographic or contextual determinants, such as socioeconomic status, may shape tumor presentation, treatment allocation, and survival in glioma populations [13].

Existing evidence on age-related glioma phenotypes derives mainly from Western European and North American registries, with limited representation of Eastern European populations. A multimodal real-world description of how age intersects with molecular, imaging, and treatment data in this setting is therefore needed [12,14].

In this context, we present an integrated, age-stratified real-world description of clinical, imaging, molecular, treatment, and survival data from consecutive adult patients with diffuse gliomas managed in a Romanian tertiary neuro-oncology center between 2021 and 2024. The aim is not to identify novel age-related biological associations, but to document how age intersects with the diagnostic, molecular, and therapeutic infrastructure of an Eastern European healthcare setting in which integrated molecular diagnostics were being progressively adopted during the study period. Specifically, we examined (i) the distribution of WHO 2021 molecular subtypes by age, (ii) the availability and uptake of individual molecular markers across the cohort, (iii) age-related patterns in surgical and adjuvant treatment allocation, and (iv) recurrence-free survival within an evolving regional standard of care.

## MATERIAL AND METHODS

### Study design

This single-center retrospective cohort study was designed and conducted at a tertiary neuro-oncology center in Romania. Reporting was performed in accordance with the Strengthening the Reporting of Observational Studies in Epidemiology (STROBE) guidelines for observational studies [15].

### Study population

Consecutive adult patients diagnosed with histologically confirmed diffuse gliomas between 2021-2024 were screened for eligibility. Tumors were classified according to the 2021 WHO Classification of Tumors of the Central Nervous System [2].

Of the 290 consecutive adult patients with histopathologically confirmed intra-axial primary brain tumors identified in our institutional registry between 2021 and 2024, the following exclusions were applied: ganglioglioma (*n* = 4), rosette-forming glioneuronal tumor (*n* = 1), and diffuse glioneuronal tumor with oligodendroglioma-like features and nuclear clusters (*n* = 1) were excluded because they do not represent diffuse glioma histology under the WHO 2021 framework. One additional patient was excluded due to incomplete pathological diagnostic information. After these exclusions, the final analytic cohort comprised 283 patients with histologically confirmed diffuse gliomas.

The study was conducted in accordance with the Declaration of Helsinki [16] and approved by the Research Ethics Subcommittee of Carol Davila University of Medicine and Pharmacy, Bucharest, Romania (registration number 35495/04.12.2025).

### Data collection and database preparation

Clinical, imaging, histopathological, molecular, treatment, and follow-up data were extracted from institutional electronic medical records and imaging archives. Data cleaning, harmonization, and preparation were performed prior to statistical analysis using OpenRefine (Google OpenRefine, version 3.8.7.), allowing standardized variable formatting and correction of inconsistencies in categorical entries.

Clinical variables included age at diagnosis and sex. Histopathological grading was performed according to WHO 2021 criteria. Molecular markers collected, where available, included IDH mutation status, 1p/19q codeletion, alpha-thalassemia/mental retardation X-linked (ATRX) status, epidermal growth factor receptor (EGFR) amplification, and Ki-67 proliferation index. Because molecular testing was not uniformly performed for all patients, analyses involving molecular variables included only those cases for which the relevant marker was available (complete-case analysis per variable), rather than excluding all patients with any missing molecular data. Baseline and postoperative Karnofsky Performance Status (KPS) were documented prospectively in clinical practice at our institution as part of routine pre- and postoperative neurosurgical and oncology assessments and recorded as numeric scores in the patient files. For this study, KPS values were extracted retrospectively from these clinical records at two predefined time points: baseline (the assessment closest to the initial consultation) and postoperative (the first documented assessment after surgery).

Preoperative MRI examinations were performed as part of routine clinical care prior to initial neurosurgical intervention. Imaging variables for the study were extracted from the original radiology reports issued by the attending neuroradiologists at the time of diagnosis; for the purposes of this study, no centralized re-review of imaging by a study-specific neuroradiologist was performed. We did not assess inter-rater reliability, and reports were not generated under blinded conditions, as they were produced as part of standard clinical workflow with knowledge of clinical context. MRI examinations were obtained on the scanners available at the referring and treating institutions during the study period, including 1.5 T and 3 T systems; standard glioma protocols included pre- and post-contrast T1-weighted sequences, T2-weighted and FLAIR sequences, diffusion-weighted imaging, and, when available, susceptibility-weighted imaging and dynamic susceptibility contrast perfusion imaging. Operational definitions extracted from the reports were as follows: (i) contrast enhancement was recorded as present when any focal post-contrast T1 signal increase was described within the tumor, with ring enhancement classified separately as a peripheral rim of enhancement around a non-enhancing center; (ii) necrosis was recorded when the report explicitly described an intratumoral non-enhancing area with imaging characteristics of necrosis (T1 hypointensity, T2 hyperintensity, absent diffusion restriction, and no internal enhancement); (iii) the T2/FLAIR mismatch sign was recorded when the report explicitly described homogeneous T2 hyperintensity with a relatively hypointense FLAIR signal in a large portion of the tumor; (iv) edema, midline shift, susceptibility hemorrhage on susceptibility-weighted imaging (SWI), and diffusion restriction were extracted as reported, dichotomised as present or absent; (v) relative cerebral blood volume (rCBV) parameters were extracted from perfusion reports as documented, with rCBVmax and rCBV trend categorised as reported (normal, mildly increased, increased, highly increased). Perfusion imaging was available in a small subset of the cohort (*n* = 24 for rCBVmax), which we acknowledge as a substantive limitation that constrains inference from perfusion-related variables. Because imaging variables were derived from clinical reports rather than from a standardized retrospective re-review, the present study cannot quantify inter-rater agreement, and individual imaging variables should be interpreted accordingly.

Treatment variables included extent of surgical resection, administration of radiotherapy, temozolomide use, and number of temozolomide cycles.

### Handling of missing data

Missing data were handled using a complete-case analysis per variable. For each molecular or imaging marker, only patients with an available result were included in the corresponding analysis; patients with missing values for that specific variable were excluded from the denominator rather than imputed. This approach reflects the real-world availability of molecular testing during the study period, which evolved as integrated molecular diagnostics were progressively adopted at our institution. The total number of patients with available data for each variable (*N*) is reported explicitly in all tables. Percentages within each age group were calculated relative to the number of tested patients in that group, not relative to the overall cohort. No imputation was performed. We acknowledge that complete-case analysis may introduce selection bias if missingness is related to patient or tumor characteristics, and this limitation is addressed in the Discussion.

Variable-level availability is reported explicitly in the *N* column of Tables 1–4. Missingness was driven by progressive institutional adoption of integrated molecular testing, histology-guided targeted testing for rare markers (1p/19q, EGFR, MGMT, TERT, CDKN2A/B, H3K27M), and uneven access to perfusion MRI across referring centers, rather than by tumor outcomes; complete-case analysis per variable was therefore used without imputation.

**Table 1:** Baseline demographic and clinicopathological characteristics according to age group

Variables	*N*	Age <40 y (*n* = 70)	Age 40-60 y (*n* = 125)	Age >60 y (*n* = 88)	*P* value
**Gender**	283				0.4
F		32 (46%)	69 (55%)	44 (50%)	
M		38 (54%)	56 (45%)	44 (50%)	
**Diagnosis type**	283				<0.001
ASTRO		60 (86%)	42 (34%)	7 (8.0%)	
GBM		4 (5.7%)	58 (46%)	74 (84%)	
OLIGO		6 (8.6%)	25 (20%)	7 (8.0%)	
**Diagnosis grade**	283				<0.001
CNS WHO grade 2		45 (64%)	39 (31%)	2 (2.3%)	
CNS WHO grade 3		16 (23%)	23 (18%)	9 (10%)	
CNS WHO grade 4		9 (13%)	63 (50%)	77 (88%)	
**KPS at diagnosis**	224				<0.001
50		0 (0%)	0 (0%)	2 (3.0%)	
60		0 (0%)	2 (2.1%)	2 (3.0%)	
70		0 (0%)	0 (0%)	1 (1.5%)	
80		0 (0%)	6 (6.2%)	13 (19%)	
90		13 (22%)	28 (29%)	26 (39%)	
100		47 (78%)	61 (63%)	23 (34%)	

Values are presented as number (percentage). Comparisons across age groups were performed using the Pearson chi-square test or Fisher’s exact test for categorical variables. A two-sided *P* value < 0.05 was considered statistically significant. Abbreviations: WHO, World Health Organization; KPS, Karnofsky Performance Status; ASTRO, astrocytoma; GBM, glioblastoma; OLIGO, oligodendroglioma.

**Table 2:** Molecular characteristics according to age group

Variables	*N*	Age <40 y (*n* = 70)	Age 40-60 y (*n* = 125)	Age >60 y (*n* = 88)	*P* value
**IDH mutant**	244	54 (89%)	55 (50%)	14 (19%)	<0.001
**1p19q deletion**	66	7 (29%)	18 (53%)	4 (50%)	0.2
**ATRX**	190	36 (71%)	17 (21%)	3 (5.1%)	<0.001
**p16**	15	3 (33%)	1 (20%)	1 (100%)	0.5
**CDKN2A/B**	6	1 (20%)	0 (NA)	1 (100%)	0.3
**TERT**	6	0 (0%)	1 (50%)	1 (50%)	>0.9
**EGFR**	22	0 (0%)	8 (89%)	5 (56%)	0.008
**KI67**	233	9 (16%)	46 (44%)	40 (57%)	<0.001
**H3 27M**	7	2 (40%)	1 (100%)	0 (0%)	>0.9

Abbreviations: GBM, glioblastoma; IDH, isocitrate dehydrogenase; WHO, World Health Organization; ATRX, alpha-thalassemia/mental retardation syndrome X-linked; MGMT, O6-methylguanine-DNA methyltransferase. Note: Values are presented as the number of positive cases (percentage of tested cases within each age group). N indicates the total number of patients with available data for the corresponding marker across the entire cohort. Percentages were calculated per age group relative to tested patients only, not relative to the full age-group population. Missing data reflect the partial availability of molecular testing during the study period.

**Table 3:** Preoperative imaging characteristics according to age group

Variables	*N*	Age <40 y (*n* = 70)	Age 40-60 y (*n* = 125)	Age >60 y (*n* = 88)	*P* value
**Lesion Enhancement**	73	12 (63%)	15 (58%)	26 (93%)	0.009
**Ring Enhancement**	73	4 (21%)	3 (12%)	6 (21%)	0.6
**Diffusion Restriction**	67	9 (50%)	6 (25%)	11 (44%)	0.2
**Necrosis**	73	7 (37%)	6 (23%)	18 (64%)	0.008
**T2/FLAIR Mismatch**	69	3 (16%)	0 (0%)	0 (0%)	0.018
**Edema**	72	13 (68%)	22 (88%)	24 (86%)	0.3
**Midline shift**	68	6 (32%)	5 (23%)	11 (41%)	0.4
**Hemorrhage SWI**	72	9 (47%)	12 (48%)	14 (50%)	>0.9
**rCBV mean increase**	20	2 (29%)	2 (50%)	7 (78%)	0.2
**rCBV max increase**	24	0 (0%)	3 (43%)	9 (82%)	0.003
**rCBV trend**	39				0.3
Highly increased		0 (0%)	0 (0%)	2 (11%)	
Increased		6 (50%)	4 (44%)	12 (67%)	
Normal		6 (50%)	5 (56%)	4 (22%)	
**Perfusion pattern**	38				0.7
Focally hyperperfused		5 (45%)	4 (50%)	11 (58%)	
Low/decreased		1 (9.1%)	0 (0%)	0 (0%)	
Mildly increased		0 (0%)	0 (0%)	2 (11%)	
Normal		5 (45%)	4 (50%)	6 (32%)	

Abbreviations: T2, T2-weighted imaging; FLAIR, fluid-attenuated inversion recovery; SWI, susceptibility-weighted imaging; rCBV, relative cerebral blood volume.

**Table 4:** Treatment characteristics and postoperative functional status according to age group

Variables	*N*	Age <40 y (*n* = 70)	Age 40-60 y (*n* = 125)	Age >60 y (*n* = 88)
**Surgical Procedure (GTR)**	185			
GTR100		20 (42%)	44 (53%)	31 (57%)
GTR80		7 (15%)	6 (7.2%)	0 (0%)
GTR85		1 (2.1%)	4 (4.8%)	0 (0%)
GTR90		11 (23%)	15 (18%)	8 (15%)
GTR95		9 (19%)	14 (17%)	15 (28%)
**Post-surgical KPS**	253			
40		0 (0%)	0 (0%)	1 (1.3%)
50		0 (0%)	1 (0.9%)	1 (1.3%)
60		0 (0%)	4 (3.6%)	4 (5.3%)
70		2 (2.9%)	2 (1.8%)	3 (4.0%)
80		2 (2.9%)	4 (3.6%)	12 (16%)
90		8 (12%)	32 (29%)	31 (41%)
100		56 (82%)	67 (61%)	23 (31%)

Abbreviations: GTR, gross total resection; KPS, Karnofsky Performance Status.

### Age stratification

Patients were stratified into three predefined age groups: <40 years, 40–60 years, and >60 years. Age group thresholds were defined a priori to reflect clinically meaningful distinctions between younger adult, intermediate, and elderly populations. These thresholds are consistent with age stratifications used in major epidemiological registries and genomic studies. The Central Brain Tumor Registry of the United States (CBTRUS) statistical report documented distinct incidence patterns of diffuse glioma subtypes across these age intervals [4], while integrated molecular analyses by Ceccarelli *et al*. demonstrated that IDH-mutant tumors predominate in younger adults, with a marked shift toward IDH-wildtype glioblastoma after the fifth decade [17]. The European Association of Neuro-Oncology (EANO) guidelines similarly recognize age as a key clinical variable influencing diagnostic workup and therapeutic planning [1]. These cutoffs thus approximate recognized biological transitions in diffuse glioma epidemiology.

### Outcome definition

Recurrence-free survival (RFS) was defined as the interval from initial surgical resection (or, for biopsy-only cases, histopathological diagnosis) to the first documented tumor recurrence or progression. Recurrence was adjudicated at the time of clinical care by the institutional multidisciplinary neuro-oncology board using imaging criteria consistent with the Response Assessment in Neuro-Oncology (RANO) framework: new measurable contrast-enhancing lesions, >25% increase in the sum of perpendicular diameters of pre-existing enhancing lesions, or significant T2/FLAIR progression in predominantly non-enhancing tumors; pseudoprogression was excluded by serial imaging at 6–12 weeks per institutional practice. Clinical deterioration attributable to tumor progression was treated as an event only when confirmed on subsequent imaging. Patients without documented recurrence were censored at last follow-up. A structured retrospective re-adjudication using full RANO 1.1 criteria was not performed; we therefore use the term “recurrence-free survival” throughout.

### Statistical analysis

Statistical analysis was performed using the free software R (version R 4.5.1). Continuous variables were summarized as medians with interquartile ranges; categorical variables as counts and percentages. Age was analyzed both as a continuous variable and categorized into three a priori defined ordinal groups (<40, 40–60, >60 years). Between-group comparisons for categorical variables were performed using Pearson’s chi-square test or Fisher’s exact test, as appropriate given expected cell counts.

Recurrence-free survival (RFS) was estimated using the Kaplan-Meier method, stratified by age groups (<40 years, 40–60 years, >60 years), and differences between survival curves were assessed using the log-rank test.

We also used suggestive graphical representations in the statistical analysis: (i) bar charts illustrating the frequency distribution of categorical dependent variables in predefined age categories; (ii) a heat map summarizing the distribution of preoperative imaging characteristics by age group and gender, with color intensity reflecting the proportion of cases in which a particular characteristic was present; and (iii) Kaplan-Meier survival curves illustrating RFS stratified by age group.

Statistical significance was set at a two-sided α = 0.05. No adjustment for multiple comparisons was applied given the exploratory nature of the analysis; *P* values should therefore be interpreted as descriptive.

R Core Team. R: A language and environment for statistical computing. Vienna (Austria): R Foundation for Statistical Computing; 2023.

## RESULTS

### Baseline demographic and histopathological characteristics according to age group

A total of 283 patients with histologically confirmed diffuse gliomas were included in the final analysis and stratified into three age groups: <40 years (*n* = 70, referred to as Group 1 in the figures), 40-60 years (*n* = 125, Group 2), and >60 years (*n* = 88, Group 3). For consistency with figure legends produced in the original analytic environment, the labels Group 1, Group 2, and Group 3 are retained in figures and tables, with age intervals indicated alongside in every legend; in the narrative below, age intervals are used directly.

**Figure 1:**
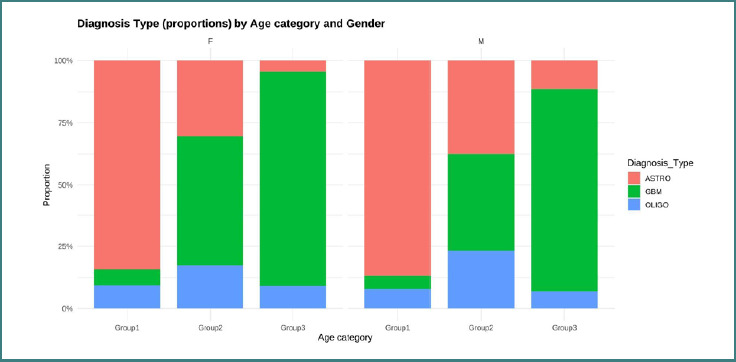
Distribution of diffuse glioma subtypes across age groups. In all figures, Group 1 corresponds to patients aged <40 years (*n* = 70), Group 2 to 40-60 years (*n* = 125), and Group 3 to >60 years (*n* = 88). Bar plot showing the proportion of astrocytoma (ASTRO), glioblastoma (GBM), and oligodendroglioma (OLIGO) within each age group.

Younger patients (<40 years) more frequently presented with lower-grade diffuse astrocytomas and oligodendrogliomas, whereas glioblastoma was markedly predominant in patients older than 60 years. The proportion of CNS WHO grade 4 tumors increased progressively with age, while CNS WHO grade 2 tumors were more common in the youngest cohort (*P* < 0.001; Figure 1).

Histopathological grading also differed significantly between groups (*P* < 0.001), demonstrating a clear age-associated shift toward higher-grade disease in elderly patients. Performance status at diagnosis varied significantly by age, with older patients having lower Karnofsky Performance Status (KPS) scores than younger individuals (*P* < 0.001). No statistically significant differences were identified regarding tumor laterality or anatomical location across age groups. Detailed baseline characteristics are presented in Table 1.

### Molecular profile across age groups

IDH mutation status differed markedly across age categories (*P* < 0.001), with IDH-mutant tumors being more frequently observed in younger patients, and IDH-wildtype tumors predominating in individuals older than 60 years.

ATRX status also varied significantly across age groups (*P* < 0.001), with ATRX loss more commonly identified in younger and intermediate-age patients than in elderly individuals. EGFR amplification was significantly associated with age (*P* = 0.008), occurring more frequently in older patients. The Ki-67 proliferation index also increased with age (*P* < 0.001), indicating higher proliferative activity in the elderly cohort. No significant differences were observed for 1p/19q codeletion, TERT promoter mutation, CDKN2A/B deletion, or H3K27M status.

**Figure 2:**
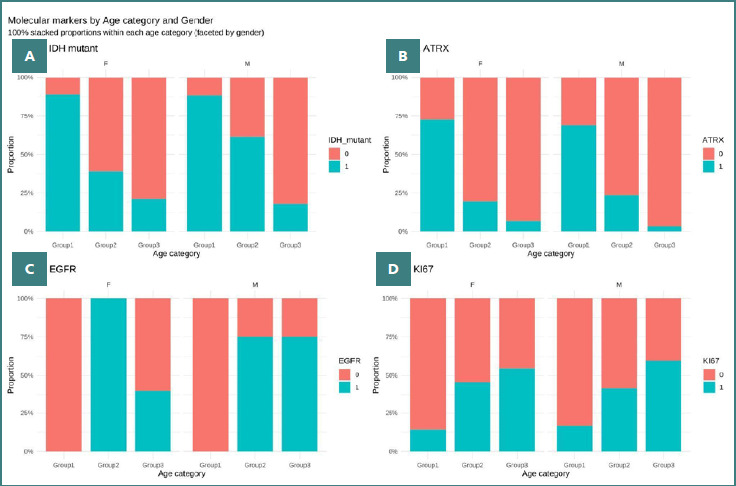
Distribution of key molecular alterations stratified by age group and sex. Age groups: Group 1 = <40 years, Group 2 = 40-60 years, Group 3 = >60 years. Denominators per marker (total tested across cohort): IDH *n* = 244, ATRX *n* = 190, EGFR *n* = 22, Ki-67 *n* = 233; percentages are computed within each age group relative to tested patients only.

Detailed molecular characteristics according to age group are presented in Table 2. The distribution of key molecular alterations, stratified by age group and sex, is illustrated in Figure 2.

### Preoperative imaging characteristics across age groups

Significant age-related differences were observed in several preoperative imaging features. Contrast enhancement patterns varied significantly across age categories (*P* = 0.009), with a higher proportion of enhancing tumors identified in patients older than 60 years. Similarly, the presence of necrosis differed between groups (*P* = 0.008), being more frequently observed in the elderly cohort. The T2/FLAIR mismatch sign showed significant variation with age (*P* = 0.018), occurring more commonly in younger patients. Perfusion parameters also differed across age groups. Relative cerebral blood volume (rCBVmax), available in a subset of patients, showed significant differences between age categories (*P* = 0.003), with higher values more frequently observed in older individuals. No significant differences were detected in tumor size, peritumoral edema, diffusion restriction, or hemorrhagic components.

**Figure 3:**
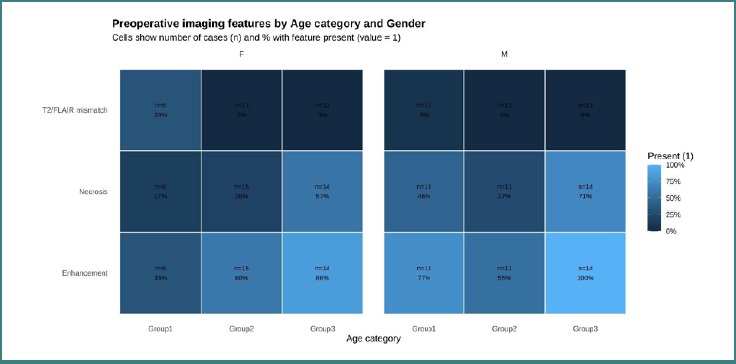
Preoperative imaging characteristics across age groups (Group 1 = <40 years, Group 2 = 40-60 years, Group 3 = >60 years). Heatmap illustrating the proportion of cases displaying contrast enhancement, necrosis, T2/FLAIR mismatch sign, and perfusion parameters within each age group. Color intensity reflects the proportion of cases in which the feature is present. Denominators per imaging variable: contrast enhancement *n* = 73, necrosis *n* = 73, T2/FLAIR mismatch *n* = 69, rCBVmax *n* = 24, rCBV trend *n* = 39, perfusion pattern *n* = 38. The small denominator for perfusion-derived metrics should be considered when interpreting between-group differences.

Detailed preoperative imaging characteristics are presented in Table 3, and the distribution of key imaging features is illustrated in Figure 3.

### Treatment patterns and surgical outcomes according to age group

The extent of surgical resection varied across age groups, although the overall distribution of gross total resection categories did not demonstrate a consistent pattern across all subgroups. However, elderly patients were less frequently treated with more aggressive surgical strategies compared with younger individuals.

Postoperative Karnofsky Performance Status (KPS) differed significantly between age categories. Lower postoperative functional scores were more commonly observed in patients older than 60 years, whereas younger patients more frequently maintained higher functional status following surgery.

Radiotherapy patterns also varied significantly across age groups. The number of radiotherapy fractions differed between cohorts (*P* = 0.005), and concomitant radiotherapy was more frequent in older patients (*P* < 0.001).

Temozolomide administration demonstrated significant age-related variation (*P* = 0.006). Younger patients were more likely to receive a higher number of temozolomide cycles (*P* < 0.001), whereas elderly patients were more likely to receive modified or shorter regimens.

### Recurrence and survival outcomes according to age

**Figure 4:**
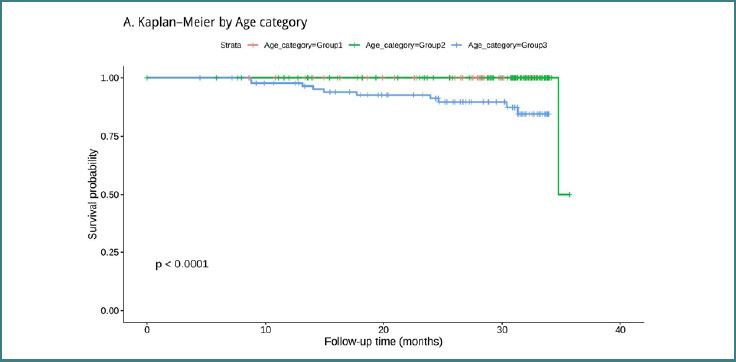
Kaplan-Meier curves of recurrence-free survival (RFS) stratified by age group (Group 1 = <40 years, *n* = 70; Group 2 = 40-60 years, *n* = 125; Group 3 = >60 years, *n* = 88). Log-rank test *P* < 0.001. The estimate is unadjusted; see Limitations regarding the absence of multivariable adjustment.

Recurrence-free survival (RFS) differed significantly across age groups. Kaplan–Meier analysis demonstrated a clear age-dependent separation of survival curves (log-rank test, *P* < 0.001; Figure 4).

Patients younger than 40 years exhibited the longest recurrence-free intervals, with the majority remaining free of documented recurrence throughout the available follow-up period; consequently, the median RFS was not reached in this subgroup. In contrast, patients aged 40–60 years and those older than 60 years reached their median RFS within the observation window, with progressively shorter intervals in the elderly cohort. This pattern is consistent with the higher prevalence of CNS WHO grade 4 and IDH-wildtype tumors in older patients. Survival curves showed early divergence within the first year following surgery, which persisted throughout follow-up. Given the relatively short study period (2021–2024) and the indolent course of IDH-mutant gliomas, which predominated in the youngest subgroup, precise median RFS estimates are not reported here, as they would be unstable and potentially misleading in groups with limited event accrual. This limitation is further addressed in the Limitations section.

## DISCUSSION

Our findings support the concept that age represents a central organizing variable in diffuse gliomas, influencing not only survival but also tumor phenotype, molecular profile, and treatment patterns. In this single-center Romanian cohort, we observed a clear age-dependent shift toward higher-grade tumors and a predominance of glioblastoma in patients older than 60 years. In contrast, younger individuals were more frequently diagnosed with lower-grade diffuse astrocytomas and oligodendrogliomas. These observations align with large population-based analyses, which have consistently demonstrated increasing incidence of IDH-wildtype glioblastoma with advancing age and a relative predominance of IDH-mutant tumors in younger adults [2,4].

The principal contribution of this cohort is contextual rather than biological. Several features of the Romanian neuro-oncology environment during the study period (2021-2024) shape the interpretation of the data. First, the WHO 2021 integrated molecular classification was implemented progressively, and the availability of individual molecular markers varied across the cohort: IDH status was documented in 244 of 283 patients (86%), ATRX in 190 (67%), Ki-67 in 233 (82%), EGFR in 22 (8%), 1p/19q codeletion in 66 (23%), MGMT in only a minority, and TERT, CDKN2A/B, and H3K27M in fewer than 10 patients each. This availability gradient is itself a feature of the regional diagnostic ecosystem rather than a per-case selection decision. Second, advanced imaging biomarkers, in particular dynamic susceptibility contrast perfusion (*n* = 24-39 depending on the parameter), were not uniformly performed across referring centers. Third, multimodal treatment intensity varied across age groups in ways that likely reflect both biological appropriateness and pragmatic constraints. We therefore present this dataset not as a vehicle for reconfirming the well-established age-IDH-grade-survival relationships, but as a transparent description of how the integrated WHO 2021 framework operates in a real-world Eastern European tertiary-care setting in transition.

The biological associations described here, namely the link between younger age, IDH-mutant tumors, and lower-grade histology, and between older age and IDH-wildtype glioblastoma, are well established in the international literature. Our contribution lies elsewhere: in documenting how these associations are reflected in a single Eastern European tertiary-care cohort in which integrated molecular diagnostics, perfusion imaging, and access to multimodal therapy progressively expanded over the study period. The documentation of treatment-pattern disparities in our cohort, including fewer temozolomide cycles and reduced access to multimodal therapy among elderly patients, may reflect not only biological constraints but also region-specific factors, such as healthcare infrastructure, reimbursement policies, and referral patterns, which remain poorly documented in Eastern European settings.

Landmark genomic analyses have demonstrated that IDH-mutant and IDH-wildtype gliomas represent separate disease entities with different evolutionary trajectories [9,17]. The age-related distribution of these subtypes in our cohort is consistent with this established biological stratification.

Older patients in our cohort had significantly lower baseline KPS scores, suggesting reduced functional reserve, consistent with prior data [5,18]. This reinforces the view that age in glioma management is a composite construct integrating both biological and patient-related factors [19]. IDH mutation status showed a clear age-dependent gradient consistent with the WHO 2021 framework [2]. The median age at diagnosis of IDH-mutant gliomas is typically two decades lower than that of IDH-wildtype glioblastoma [9,17].

IDH-mutant gliomas show a distinct epigenetic profile and lower proliferative index, contributing to their more indolent course [20,21], whereas IDH-wildtype glioblastoma frequently harbor EGFR amplification, TERT promoter mutations, and chromosome 7/10 alterations associated with rapid progression [22,23]. The age-dependent shift toward IDH-wildtype, high-grade tumors in patients over 60 years in our cohort likely reflects this molecular contrast.

MGMT promoter methylation, an established predictor of temozolomide response [24], was available in only a minority of patients during the study period and was therefore not used as a stratifying variable.

Older patients more often presented with contrast-enhancing and necrotic lesions, imaging features classically linked to high-grade IDH-wildtype tumors [25]. Elevated rCBV values were also more frequent in older patients, although the small subset with available perfusion data (*n* = 24-39 depending on the parameter) precludes firm conclusions. These observations are consistent with the broader principle that imaging phenotype tracks molecular architecture.

Elderly patients in our cohort received less intensive multimodal therapy, in line with established clinical practice patterns that balance oncological aggressiveness with frailty considerations [26]. Whether this reflects appropriate adaptation or healthcare-system constraints cannot be disentangled in the present dataset. In our cohort, the variable availability of MGMT methylation across the study period precluded meaningful stratification by this established predictive biomarker [27].

Contemporary practice emphasizes that biological age, molecular subtype, and functional status are more relevant therapeutic determinants than chronological age alone [26,27].

In the present cohort, recurrence-free survival differed significantly across age groups, with progressively shorter RFS observed in older patients. This finding is consistent with extensive population-based evidence demonstrating that advanced age is associated with inferior survival in diffuse gliomas, particularly in IDH-wildtype glioblastoma [4,26]. Because we did not perform a multivariable Cox regression in this exploratory analysis, the age-related survival differences observed here cannot be attributed to an independent effect of chronological age and should be interpreted as the cumulative reflection of age-correlated tumor grade, molecular subtype, performance status, and treatment allocation, as discussed in the Limitations. However, our findings suggest that these differences cannot be attributed to chronological age alone but reflect the cumulative effect of tumor characteristics, baseline performance status, and therapeutic allocation.

The age-dependent predominance of CNS WHO grade 4 and IDH-wildtype tumors likely represents the principal biological driver of the survival disparities observed in our dataset. At the same time, reduced postoperative functional reserve and potential limitations in treatment intensity among elderly patients may further contribute to shorter recurrence-free intervals. Contemporary analyses emphasize that survival in glioblastoma remains multifactorial, influenced by extent of resection, molecular markers such as MGMT promoter methylation, and multidisciplinary coordination of care [28,29].

Most large-scale survival datasets originate from Western European or North American registries. By providing integrated real-world data from a Romanian tertiary center, this study complements international trial-based evidence with region-specific descriptive context.

Taken together, these data are consistent with the view that age in diffuse gliomas functions as a composite biological and clinical variable, capturing correlated tumor, host, and treatment factors rather than acting as an independent prognostic mechanism. Integrative descriptive datasets such as ours may help calibrate expectations for real-world risk stratification.

### Limitations of the study

This study has several limitations that should be considered when interpreting its findings.

First, the retrospective, single-center design introduces inherent constraints on data completeness and generalizability. Molecular testing was not uniformly available across the cohort, reflecting the progressive adoption of integrated molecular diagnostics at our institution during the study period. A complete-case analysis per variable was therefore applied, which may introduce selection bias if missingness is non-random. Perfusion imaging was similarly available only in a subset of patients. The total number of patients with available data is reported explicitly for each variable in the corresponding tables.

Second, the analysis was designed as exploratory and descriptive, relying on univariate and bivariate comparisons stratified by age and sex, complemented by Kaplan-Meier estimates with log-rank tests. We deliberately did not fit a multivariable Cox proportional hazards model for recurrence-free survival, despite its relevance, for three reasons. First, the joint availability of all candidate covariates (age, WHO 2021 grade, IDH status, baseline KPS, and extent of resection) is restricted to a substantially smaller subset than the full cohort because molecular testing and detailed surgical documentation evolved progressively during the study period; specifically, IDH status was available in 244 patients, ATRX in 190, Ki-67 in 233, with full surgical resection categorisation in 185 and baseline KPS in 224, leaving fewer than 150 cases with simultaneous availability of all five covariates. Second, the resulting effective sample size, combined with the short observation window (2021-2024) and the fact that the median RFS was not reached in the youngest subgroup, yields a low and uneven event-per-variable ratio, making multivariable hazard estimates unstable and prone to overfitting. Third, missingness for several molecular markers is non-random (it reflects calendar-time adoption of testing rather than tumor biology), so multivariable models without imputation would propagate this selection bias, while imputation across such a heterogeneous availability gradient is itself susceptible to misspecification. We therefore report this study as a descriptive integrated snapshot. A multivariable Cox model, with multiple imputation for molecular markers and sensitivity analyses for non-random missingness, is planned for an extended cohort with longer follow-up.

Third, no formal a priori sample size calculation was performed, as the study included all consecutive eligible patients treated at our center during the study period. Although the final sample (*n* = 283) is relatively large for a single-center neuro-oncology study in this region, statistical power for subgroup analyses involving molecular markers with limited testing availability (e.g., TERT, CDKN2A/B, H3K27M) is necessarily constrained, and borderline *P* values for these variables should be interpreted with caution.

Fourth, the relatively short observation window (2021–2024) and censoring patterns preclude reliable estimation of median recurrence-free survival, particularly in the youngest subgroup, where IDH-mutant tumors with an indolent biological course predominate and median RFS typically exceeds 5–10 years. Reported findings therefore primarily reflect early and intermediate recurrence patterns rather than long-term outcomes. We used recurrence-free survival rather than RANO-adjudicated progression-free survival, as consistent retrospective application of the Response Assessment in Neuro-Oncology criteria was not feasible across all cases; recurrence was defined as documented radiological or clinical progression recorded in institutional medical records. Overall survival was not analyzed as a primary endpoint due to follow-up structure constraints.

Fifth, several sources of bias warrant consideration. Selection bias may arise from the tertiary-care setting, which likely over-represents surgically treated and molecularly profiled cases compared with the general glioma population in Romania. Referral bias may further affect the distribution of tumor grades and molecular subtypes, as patients with more complex or atypical presentations may be preferentially referred to our center. Information bias related to retrospective imaging and pathology review was mitigated by using routine clinical reports issued at the time of diagnosis, although inter-reader variability cannot be excluded, and inter-rater agreement was not formally quantified, as imaging variables were extracted from clinical reports rather than from a study-specific centralized re-review. Finally, the evolving availability of molecular testing across the study period may have introduced temporal bias in marker detection rates.

Despite these limitations, the study has notable strengths: a relatively large single-center cohort for a biologically heterogeneous disease, consistent pathological classification according to the 2021 WHO criteria, and the integration of clinical, imaging, molecular, treatment, and survival data within a single age-stratified analytical framework. To our knowledge, this represents the first such integrative characterization of adult diffuse gliomas reported from a Romanian neuro-oncology center.

## CONCLUSION

In this single-center Romanian cohort, age was associated with tumor phenotype, molecular architecture, functional status, imaging characteristics, and recurrence-free survival among diffuse gliomas, consistent with the international literature. Because the analysis was descriptive and did not include multivariable adjustment, age should not be interpreted here as an independent prognostic determinant but rather as a composite marker capturing correlated biological and clinical variables. Older patients demonstrated a higher prevalence of CNS WHO grade 4 and IDH-wildtype tumors, more aggressive imaging characteristics, reduced performance status at presentation, and shorter recurrence-free intervals.

By integrating clinical, imaging, molecular, treatment, and survival data within an age-stratified framework, this study provides real-world evidence from an underrepresented Eastern European population. Such datasets may contribute to improved risk stratification and more nuanced, individualized management strategies across age groups in contemporary neuro-oncology practice.

## Data Availability

The data presented in this study are available upon reasonable request from the corresponding author. The data are not publicly available due to privacy and ethical restrictions related to patient confidentiality.

## Personal thanks

The authors would like to thank all clinicians, radiologists, pathologists, and allied healthcare professionals involved in the diagnosis, treatment, and follow-up of patients included in this study. We also acknowledge the institutional support that facilitated data collection and database maintenance. Finally, we are grateful to the patients whose anonymized data contributed to advancing knowledge in neuro-oncology. During the preparation of this manuscript, the authors used Claude (Anthropic) and ChatGPT (OpenAI) to assist with language editing, text structuring, and clarity improvement. The authors reviewed and edited all generated content and take full responsibility for the final version of the manuscript.
